# Transcript Profiles of Stria Vascularis in Models of Waardenburg Syndrome

**DOI:** 10.1155/2020/2908182

**Published:** 2020-08-01

**Authors:** Linjun Chen, Lin Wang, Lei Chen, Fangyuan Wang, Fei Ji, Wei Sun, Hui Zhao, Weiju Han, Shiming Yang

**Affiliations:** ^1^College of Otolaryngology Head and Neck Surgery, Chinese PLA General Hospital, Beijing, China; ^2^Nursing Department, Hainan Hospital of Chinese PLA General Hospital, Sanya 572013, China; ^3^Chongqing Academy of Animal Science, Chongqing 402460, China; ^4^National Clinical Research Center for Otolaryngologic Diseases, Beijing, China; ^5^Key Lab of Hearing Science, Ministry of Education, China; ^6^Beijing Key Lab of Hearing Impairment for Prevention and Treatment, Beijing, China; ^7^Department of Communicative Disorders and Sciences, Center for Hearing and Deafness, The State University of New York at Buffalo, Buffalo, New York, USA

## Abstract

**Background:**

Waardenburg syndrome is an uncommon genetic condition characterized by at least some degree of congenital hearing loss and pigmentation deficiencies. However, the genetic pathway affecting the development of stria vascularis is not fully illustrated.

**Methods:**

The transcript profile of stria vascularis of Waardenburg syndrome was studied using Mitf-M mutant pig and mice models. Therefore, GO analysis was performed to identify the differential gene expression caused by Mitf-M mutation.

**Results:**

There were 113 genes in tyrosine metabolism, melanin formation, and ion transportations showed significant changes in pig models and 191 genes in mice models. In addition, there were some spice's specific gene changes in the stria vascularis in the mouse and porcine models. The expression of tight junction-associated genes, including Cadm1, Cldn11, Pcdh1, Pcdh19, and Cdh24 genes, were significantly higher in porcine models compared to mouse models. Vascular-related and ion channel-related genes in the stria vascularis were also shown significantly difference between the two species. The expression of Col2a1, Col3a1, Col11a1, and Col11a2 genes were higher, and the expression of Col8a2, Cd34, and Ncam genes were lower in the porcine models compared to mouse models.

**Conclusions:**

Our data suggests that there is a significant difference on the gene expression and function between these two models.

## 1. Background

Waardenburg syndrome (WS) is a rare genetic condition characterized by at least some degree of congenital hearing loss and pigmentation deficiencies [1]. WS has more than 20 mutations in the Mitf allele [2, 3], including Mitf^mi-vga9^, Mitf^mi-bw^, and Mitf^mi-ce^, which have been identified to cause hearing loss and changes in pigmentation. Although the mouse model is widely used in disease phenotypes and pathogenic mechanisms of deafness-related research [4–8], many shortcomings have also been found in studying human genetic diseases. As there is a tremendous revolutionary difference between mouse and human, it may cause a huge biological difference in anatomy, energy metabolism, and auditory perception [9, 10]. For example, the developmental patterns of auditory organs are different in mice and humans: human's hearing developed before birth while mouse's hearing did not fully developed until two weeks after birth [11]. Some studies [4, 12] found that human embryonic developmental diseases are difficult to be replicated in some of the mouse models. Therefore, different animal models, such as cattle [13], horses [14], dogs [15, 16], and pigs [17] were also necessary to be used to study genetic diseases. Pigs are precocial species with fully developed auditory system at birth. Recent studies also found the cochlear anatomy is very similar to human [18–21]. As pigs are large-scale animals with high reproductive efficiency and economical convenience, it is a good model for study auditory genetic diseases [22–24].

The stria vascularis plays an important role in maintaining the cochlear endolymphatic potentials (EP) which is essential for the mechanical electrical conduction of the hair cells [25–29]. The stria vascularis is composed with the macrophage-like melanocytes, which also called the intermediate cells [28, 30, 31]. The potassium ions in the Scala media are produced by the intermediate cells and several potassium channels and transporters in the lateral wall, such as KCNQ1/KCNE1, KCNQ4, KCNN2, KCNJ10, and SLC12A2, are also involved in maintaining the endolymphatic potentials [26, 32–34]. When malfunction of stria vascularis will result in hearing loss [35]. For example, Marcus [36] reported that Kcnj10 knockout can decrease EP value from +80 mV to 1 mV, and the K^+^ concentration decrease from 110 mM to 60 mM in their mouse models. In our previous studies, we found that Mitf-M knockout can decrease the EP to 18 mV in the mouse model [37]. In the Mitf knockout pig model, we found that the Mitf mutation caused the value of EP dropped from +78 mV to +3 mV, which was lower than the mouse model [18]. In the wild type pig, the potassium concentration in the endolymph was 142 mM higher than those in the perilymph. Our previous study found the potassium concentration dropped to 0 mM in the Mitf mutant pigs [18]. We expect that there may be different genes in maintaining the EP in mice and pigs, and the mutation of Mitf gene may cause a different change in potassium channels. To answer these questions, this study attempted to detect changes in the genetic profiles of these two species caused by Mitf-M gene mutation in RNA transcriptome level. As most of the current researches only use mouse models, this paper will further detect the RNA transcriptome difference in the stria vascularis between the large animals and mouse models.

## 2. Results

### 2.1. Gene Expression Changes Caused by Mitf-M Mutation

**Figure 1:**
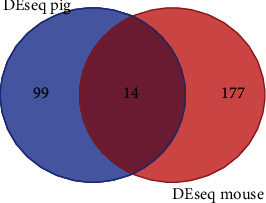
Venn diagram of DEGs in Mitf-M mutant and normal pigs/mice. The left circle represents the DEGs in the Mitf-M mutant and normal pigs; the right circle represents the DEGs in the Mitf-M mutant and normal mice. The middle part represents the DEGs in the Mitf-M mutant and normal pigs/mice.

The activation of different genes in the stria vascularis of pigs and mice caused by the Mitf-M mutation compared to their W/T controls were screened using the DESeq package software. The conditions for screening differential genes were corrected *p* value <0.05. The intersections of the differential genes in the pigs and mice were obtained using the Venn diagrams. The results were shown in Table 1. There were 14 common differential genes between the Mitf mutant animals and the controls. There were 177 specific differential genes in mouse model and 99 specific differential genes in pig models (Figure 1).

**Figure 2:**
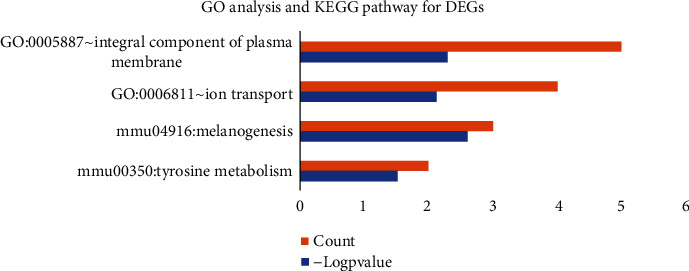
List of GO-terms with significant enrichment of DEGs. From top to bottom, the enrichment value decreases. The red *X*-axis indicates the number of unigenes in a category; the blue *X*-axis indicates the value of log_2_ (*p* value) in corresponding category. The *Y*-axis indicates the specific category.

The GO analysis and the KEGG pathway analysis were performed on the David Database (adjusted *p* value <0.05). The results were shown in Figure 2. The main pathway caused by Mitf mutation was the KEGG pathway, enriched in the tyrosine metabolism (mmu00350) and the melanogenesis pathway (Melanogenesis, mmu04916). The GO analysis was mainly enriched in the biological process of ion transport (ion transport, GO: 0006811) and the integral component of plasma membrane (GO: 0005887) (Figure 2).

### 2.2. Stria Vascularis Specific Ion Transport-Related Gene Analysis

**Figure 3:**
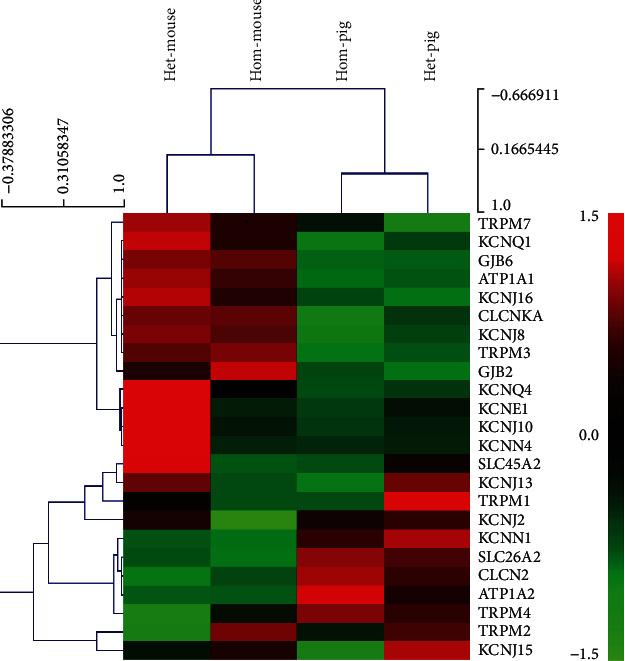
Ion channel relevant genes for cluster analysis heat map. Each column represents an experimental sample. Hom-mouse and het-mouse represent Mitf-M knockout mice and normal control mice. Hom-pig and het-pig represent Mitf-M mutant pigs and normal control pigs. Each row represents a gene. Different expressions are shown in different colors: red represents more expression and green represents less expression.

Ion transport-related genes were extracted from RNA transcriptome data from the normal and Mitf-m mutant pigs and mice samples for cluster analysis. The results showed many ion transport-related genes were highly expressed in both species through MeV cluster analysis (Figure 3). The Mitf mutation was coaffected with *Trpm1*, *Kcnj13*, and *Slc45a2* genes in both species. There were significant differences in ion channel regulation between pigs and mice. The expression of *Kcnn1*, *Clcn2*, and *Trpm4 genes* was higher in pigs than those genes in the mice, whereas the expression of *Trpm7*, *Kcnq1*, and *Kcnj8* genes was found higher in mice compared to the pigs.

### 2.3. The Specific Tight Junction-Associated Genes in the Stria Vascularis

**Figure 4:**
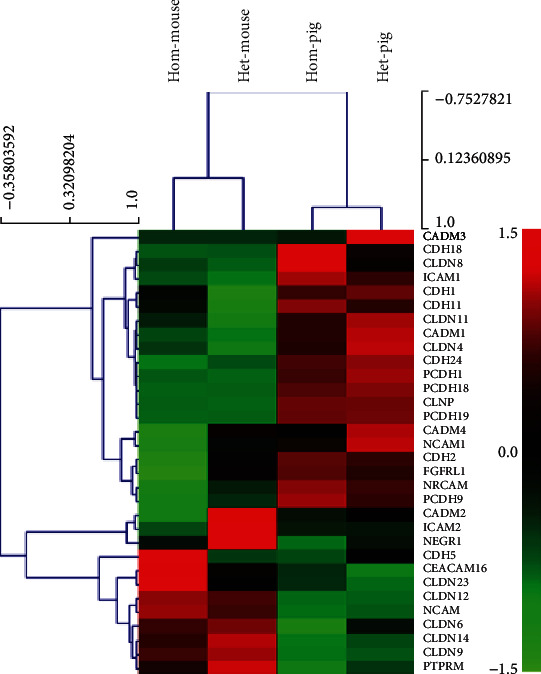
Tight junction relevant genes for cluster analysis heat map. Hom-mouse and het-mouse represent Mitf-M knockout mice and normal control mice. Hom-pig and het-pig represent Mitf-M mutant pigs and normal control pigs.

**Figure 5:**
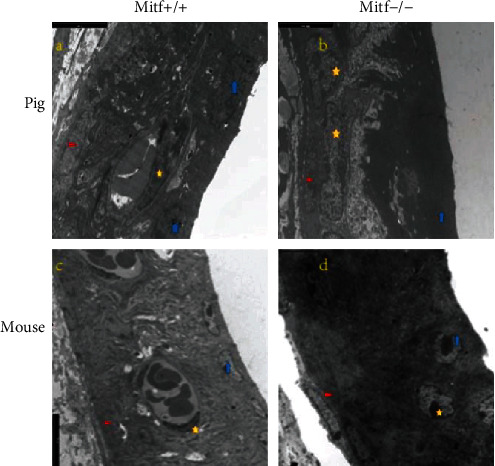
The SEM shows the cochlear stria vascularis of Mitf mutant and normal pigs/mice. (a) A normal pig's stria vascularis. (b) The stria vascularis from a mutant pig. (c) The stria vascularis from a normal mouse. (d) The stria vascularis from a mutant mouse. The red triangle marks the basal cells, the yellow pentagon marks the middle cells, and the blue arrow marks the marginal cells. The scale bar is 5 *μ*m.

The tight junction-associated genes were extracted from the RNA transcriptome data from the Mitf mutant and normal pigs/mice for cluster analysis. The expression of tight junctions in the stria vascularis of the two species was different (Figure 4). The *Cadm1*, *Cldn11*, *Pcdh1*, *Pcdh19*, and *Cdh24* genes expressed higher in pigs compared to those genes in mice, whereas *Ncam*, *Cldn6*, *Cldn9*, and *Cldn14* genes expressed higher in mice compared to pigs. And it was found that both the structures of the stria vascularis in two groups were intact. As the marginal nuclei and the cell connections were intact. The three layers of cells were obvious, and the basal cells were closely connected (Figure 5).

### 2.4. Stria Vascularis Specific Vascular Development-Related Genes

**Figure 6:**
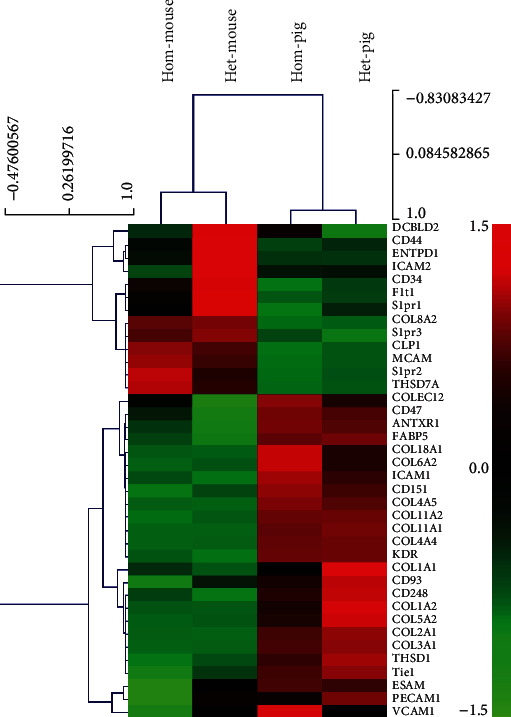
Heat analysis of clustering analysis of genes associated with specific vascular endothelial cell in normal and Mitf mutant pigs/mice (hom-mouse and het-mouse represent Mitf-M knockout mice and normal mice, hom-pig and het-pig represent Mitf-M mutant pigs and normal pigs).

Extracted vascular development-related genes from the RNA transcriptome data of the Mitf mutant and normal pigs/mice were used for cluster analysis. There was a significant difference in the vascular developmental genes in the stria vascularis between these two species (Figure 6). The *Col2a1*, *Col3a1*, *Col11a1*, and *Col11a2* genes expressed higher in the pigs than the mice, whereas the *Col8a2*, *Cd34*, and *Ncam* genes expressed higher in the mice compared to the pigs.

## 3. Discussion

This study reviewed the changes of Mitf-M mutation on gene expression in the cochlea. Mitf has many subtypes [38], in which type M is specifically expressed in melanocytes [39], by direct association with related pigmentases such as tyrosinase (Tyr), dopachrome tautomerase (Dct), endothelin receptor type B (Ednrb), and solute carrier family 45 member 2 (Slc45a2), regulating the survival, migration, and differentiation of melanocytes [40]. Among them, Mitf-M gene [38, 39] plays a key role in regulating tyrosine metabolic pathway and melanin production, mainly regulating downstream pigment-related enzymes such as Tyr, Dct, and Tyrp1. Mitf-M also controls cytoskeleton and intercellular tight protein to regulate morphology and migration of melanocytes. In this study, we found the main gene pathway caused by the Mitf-M mutation is on ion transport pathway, including the tyrosine, acid metabolism, and melanin formation pathways, in the cochlear stria vascularis of both mice and pigs. Our data are consistent with previous reports [39].

Our previous studies reported that the Mitf-M gene mutation in the Waardenburg 2A pigs and mice through a deletion of the Mitf-M genes, which caused melanocytes failed to migrate to the cochlear stria vascularis. It can cause drops of EP and damages of cochlear hair cells. In this study, we also identified a significant decrease of the K^+^ channel-associated genes, i.e., *Trpm1*, *Kcnj13*, *Slc45a2*, and *Kcnj10*. From our RNA-seq sequencing analysis, *Clcnka* and *Kcnj15* genes showed a significant difference in pig models, whereas the *Kcnq4*, *Kcnn4*, *Kcne1*, and *Kcnj2* genes are affected mainly in the mouse models. KCNJ13 (KIR7.1) and KCNJ10 (KIR4.1) belong to the inward rectifier potassium channel category. KCNJ10 is known as the key channel of potassium transport. It has been deeply studied in deafness-related diseases, and its deletion can lead to the reduction of EP and potassium ion concentration [36, 41–45]. However, Kcnj13, Trpm1, and Slc45a2 were rarely reported in auditory researches. In addition, TRPM1 is a nonselective voltage-gated cation channel in the transient receptor potential (TRP) family, and the *Mitf* mutation can lead to the deletion of *Trpm1* [46]. SLC45A2 is a cross-mediated melanin synthesis in membrane transporter [47], which is regulated by *Mitf* via the cAMP pathway through *Tyr* and *Dct* genes, the major pigment-related genes [48, 49].

The stria vascularis transcriptome data of the two species indicated that Mitf-M played an important role in regulating the expression of the *Trpm1*, *Kcnj13*, *Slc45a2*, and *Kcnj10* genes in the stria vascularis. In both species, Mitf-M may play an important role in the auditory development and maintain the EP in the cochlea. Although there were huge biological differences between pigs and mice, we found that common gene changes in both species caused by Mitf-M. Mitf-M mutation induced a significant change in *Clcn2*, *Kcnn1*, and *Trpm4* genes in the both models. CLCN2 [34] is an important component of chloride channel, which coordinates potassium and chloride exchanges. The function of KCNN1 has not been reported in the inner ears. KCNN1 belongs to the calcium ion-mediated potassium channels and plays an important role in the regulation of neural inflammation and nerve aging by microglia [50]. In mice, *Kcnq1*, *Trpm7*, and *Kcnj8* were significantly affected. KCNQ1 is a calcium ion-dependent potassium channel [45]. When Kcnq1 is deleted, it will cause degeneration of the outer hair cells, which is clinically characterized as Jervell and Lange-Nielsen syndrome, one condition that causes profound hearing loss from birth and a disruption of the heart's normal rhythm. KCNE1 and KCNQ1 are important potassium-secreting channels in the stria vascularis marginal cells [26, 45, 51]. KCNE1 regulates KCNQ1 expression and increases ion transport [52].

The tight junctions and vascular endothelial cells are important components of the blood labyrinth barrier as well as ion channels [30, 53–57]. Our cluster analysis of the RNA transcriptome data from both pigs and mice showed that the tight junctions were significantly different in the stria vascularis of these two species. The expression of *Cadm1*, *Cldn11*, *Pcdh1*, *Pcdh19*, and *Cdh24* was found higher in pigs compared to mice, whereas the expression of *Ncam*, *Cldn6*, *Cldn9*, and *Cldn14* genes were higher in mice compared to pigs. Cluster analysis of vascular-related genes revealed that it was significantly different in the stria vascularis of the two species. The higher expression in pigs is *Col2a1*, *Col3a1*, *Col11a1*, and *Col11a2*, whereas the expression of *Col8a2*, *Cd34*, and *Ncam* genes was higher in mice compared to pigs. These results may reveal that the two animals may invoke different genes to regulate the tight junction, just as the ion channels. The differences between the two species' evolutionary relationship, living habits, and anatomy may result in significant differences in these gene expressions [56, 58, 59]. It is more suitable to choose animal model closer to humans to study auditory related diseases.

In summary, we show that by leveraging RNA-seq for the analysis of the stria vascularis of the WS models, it helps to understand the regulatory mechanisms related to the loss of EP and deafness. These data provide insight into ion channel-defining genes and illustrate the possible genes associated with the WS hearing loss. These results may make a fundamental effect on the gene therapy, which used to rescue the elapse of the endocochlear potential in the stria vascularis.

## 4. Conclusion

Our research reveals that there exists a huge difference on the gene expression and function between these two models. According to the different expression in the genetic profiles of these two species caused by Mitf-M gene mutation in RNA transcriptome level, there may be different genes transcript pathway caused by mitf mutation in regulating the potassium channels in mice and pigs. And this transcriptome data may provide a basis for the gene therapy in treating the Waardenburg syndrome.

## 5. Material and Methods

### 5.1. Animals

Both Mitf mutant and normal pigs and mice have been used in this experiment. The generation of the Mitf mutant pigs and mice have been described in our previous publications [17, 37]. The experimental protocols were approved by the ethics committee of the Chinese PLA Medical School.

### 5.2. RNA Isolation from Stria Vascularis Tissue

Tissues of the stria vascularis of pigs were obtained from four normal pigs and four Mitf mutant pigs at E85 of embryonic stage as previous study described [17]. The tissues of stria vascularis of mice were obtained from ten normal mice and ten Mitf mutant mice at postnatal 30 days as previous studies described [7, 37]. The total RNA of these tissues was extracted separately using Trizol reagent (Invitrogen, CA, USA) following the manufacturer's protocol. The quantity, purity, and integrity of the collected total RNA were analyzed with NanoPhotometer® spectrophotometer (IMPLEN, CA, USA), a Bioanalyzer 2100, and RNA Nano 6000 Assay Kit (Agilent, CA, USA). Approximately, 4 *μ*g of total RNA was used for the RNA sample preparations as previous studies described [60, 61].

### 5.3. Library Construction and Sequencing

The NEBNext® Ultra TM RNA Library Prep Kit for Illumina® (NEB, USA) was used for the sequencing library preparation, which was conducted with an Illumina HiSeq TM 2000 system following the manufacturer's recommended protocol (Illumina Company Ltd, San Diego, CA, USA) as previous studies described [61, 62].

### 5.4. RNA-Seq Reads Mapping

The reference genome and gene model annotation files were obtained from Genome Web (http://asia.ensembl.org/index.html). The index of the reference genome was built using Hisat2 software (v2.0.5), and the paired-end clean reads were aligned to the reference genome. A database of potential splice junctions was built and confirmed by comparing the previously unmapped reads against the database of putative junctions. The aligned read files were processed by Cufflinks software, which used the normalized RNA-seq fragment counts to measure the relative abundances of the transcriptome. The unit of measurement was fragmented per kilobase of exons per million fragments mapped (FPKM). Reads were mapped into the mouse NCBIM38 (ensemble release 68) and the Sus scrofa 11.1 (sus scrofa ensembl release 94) using default options.

### 5.5. Gene Ontology (GO) and Pathway Enrichment Analysis of DEGs

Differential expression analysis of Mitf-M mutant and normal pigs/mice were performed using the DEseq R package [63]. Using the adjusted *p* values 0.05 and setting the absolute fold change of 2 as the threshold for significantly differential expression. Using Gene Ontology (GO) and KEGG to analyze high-throughput genome and transcriptome data in the DAVID database [64–68], which is an important online tool for these analyses. The DEGs list was uploaded to the DAVID [64] analysis tool, and *p* < 0.05 was considered statistically significant. The DEGs was uploaded to the MeV software (https://sourceforge.net/projects/mev-tm4/) to get the relevant heat map.

### 5.6. Selecting Deafness Gene of SV Transcriptomes from RNA-Seq Data

In obtaining our data for known deafness genes, we used a database of known deafness genes from these sources: (1) Hereditary Hearing Loss homepage [69] and (2) Hereditary hearing loss and deafness overview [27, 45].

### 5.7. The Transmission Electron Microscopy (TEM)

To prepare samples for TEM examination, the stria vascularis were washed with 0.1 M PBS and then postfixed in 1% osmium tetroxide and then dehydrated by a series of ethanol before embedded in plastic Agar 100 resin. After polymerization, the stria vascularis was cut into ultrathin sections (3 *μ*m), stained with toluidine blue, were mounted on 0.7% formvar coated copper grids, contrasted by 0.5% uranyl acetate and lead citrate, then examined under a transmission electron microscopy (Philips Tecnai10) [70].

## Figures and Tables

**Table 1:** The common DEGs in pigs/mice with/without Mitf-M mutation.

Gene_Name	hom_pig	het_pig	MM_mouse	WW_mouse
*Tyr*	0.014667	9.40833	0.00233	37.4824
*Emilin2*	65.6574	36.3192	52.3576	20.7289
*Gsn*	199.663	289.825	64.9488	223.727
*Dct*	1.09829	135.912	0.00277	774.431
*Gpnmb*	2.22801	47.8019	1.14682	86.5487
*Ednrb*	1.19691	12.2565	1.66075	26.9099
*Ucma*	198.465	285.077	256.207	523.427
*Slc45a2*	0.793729	24.5716	3.9E-07	45.1788
*Tspan10*	3.93361	18.2708	0.036796	14.1373
*Clca2*	68.3216	50.1806	1.59181	0.120315
*Kcnj10*	4.4472	14.4644	16.594	111.93
*Trpm1*	0.009755	14.7644	0.035483	6.75993
*Plp1*	0.215656	4.79304	7.71325	34.9961
*Kcnj13*	0.149383	14.6624	1.753	14.403

## Data Availability

Readers can access additional experimental data in optional supplementary materials.

## Supplementary Materials

Supplementary materials Deseq GO Analysis. Pig and mouse DEG.

## References

[1] Song J., Feng Y., Acke F. R., Coucke P., Vleminckx K., Dhooge I. J. (2016). Hearing loss in Waardenburg syndrome: a systematic review. *Clinical Genetics*.

[2] Hai T., Guo W., Yao J. (2017). Creation of miniature pig model of human Waardenburg syndrome type 2A by ENU mutagenesis. *Human Genetics*.

[3] Watanabe A., Takeda K., Ploplis B., Tachibana M. (1998). Epistatic relationship between Waardenburg Syndrome genes MITF and PAX3. *Nature Genetics*.

[4] Ohlemiller K. K., Jones S. M., Johnson K. R. (2016). Application of mouse models to research in hearing and balance. *Journal of the Association for Research in Otolaryngology*.

[5] Omichi R., Shibata S. B., Morton C. C., Smith R. J. H. (2019). Gene therapy for hearing loss. *Human molecular genetics*.

[6] Gao X., Tao Y., Lamas V. (2018). Treatment of autosomal dominant hearing loss by _in vivo_ delivery of genome editing agents. *Nature*.

[7] Tan F., Chu C., Qi J. (2019). AAV-ie enables safe and efficient gene transfer to inner ear cells. *Nature Communications*.

[8] Pan B., Askew C., Galvin A. (2017). Gene therapy restores auditory and vestibular function in a mouse model of Usher syndrome type 1c. *Nature Biotechnology*.

[9] Wang D. (2015). Evolution and Restoration of Structures and Functions of the Human Central Nervous System—A Review. *Journal of Neurorestoratology*.

[10] Müller U., Barr-Gillespie P. G. (2015). New treatment options for hearing loss. *Nature Reviews Drug Discovery*.

[11] Tritsch N. X., Bergles D. E. (2010). Developmental regulation of spontaneous activity in the mammalian cochlea. *The Journal of Neuroscience*.

[12] Bommakanti K., Iyer J. S., Stankovic K. M. (2019). Cochlear histopathology in human genetic hearing loss: State of the science and future prospects. *Hearing Research*.

[13] Philipp U., Lupp B., Mömke S. (2011). A MITF mutation associated with a dominant white phenotype and bilateral deafness in German Fleckvieh cattle. *PLoS ONE*.

[14] Hauswirth R., Haase B., Blatter M. (2012). Mutations in MITF and PAX3 cause "splashed white" and other white spotting phenotypes in horses. *PLoS Genetics*.

[15] Tsuchida S., Takizawa T., Abe K., Okamoto M., Tagawa M. (2009). Identification of microphthalmia-associated transcription factor isoforms in dogs. *Veterinary Journal*.

[16] Körberg I. B., Sundström E., Meadows J. R. S. (2014). A simple repeat polymorphism in the MITF-M promoter is a key regulator of white spotting in dogs. *PLoS ONE*.

[17] Chen L., Guo W., Ren L. (2016). A de novo silencer causes elimination of MITF-M expression and profound hearing loss in pigs. *BMC Biology*.

[18] Guo W., Yi H., Ren L. (2015). The Morphology and Electrophysiology of the Cochlea of the Miniature Pig. *The Anatomical Record*.

[19] Du Y., Ren L.-l., Jiang Q.-q. (2019). Degeneration of saccular hair cells caused by MITF gene mutation. *Neural Development*.

[20] Hao Q.-Q., Li L., Chen W. (2018). Key Genes and Pathways Associated With Inner Ear Malformation in SOX10 p.R109W Mutation Pigs. *Frontiers in Molecular Neuroscience*.

[21] Yi H., Guo W., Chen W., Chen L., Ye J., Yang S. (2016). Miniature pigs: a large animal model of cochlear implantation. *American Journal of Translational Research*.

[22] Ji X.-J., Chen W., Wang X. (2019). Canalostomy is an ideal surgery route for inner ear gene delivery in big animal model. *Acta Oto-Laryngologica*.

[23] An F.‐. W., Yuan H., Guo W. (2018). Establishment of a Large Animal Model for Eustachian Tube Functional Study in Miniature Pigs. *The Anatomical Record*.

[24] Lovell J. M., Harper G. M. (2007). The morphology of the inner ear from the domestic pig Sus scrofa. *Journal of Microscopy*.

[25] Dror A. A., Avraham K. B. (2010). Hearing Impairment: A Panoply of Genes and Functions. *Neuron*.

[26] Korrapati S., Taukulis I., Olszewski R. (2019). Single Cell and Single Nucleus RNA-Seq Reveal Cellular Heterogeneity and Homeostatic Regulatory Networks in Adult Mouse Stria Vascularis. *Frontiers in molecular neuroscience*.

[27] Uetsuka S., Ogata G., Nagamori S. (2015). Molecular architecture of the stria vascularis membrane transport system, which is essential for physiological functions of the mammalian cochlea. *The European Journal of Neuroscience*.

[28] Nyberg S., Abbott N. J., Shi X., Steyger P. S., Dabdoub A. (2019). Delivery of therapeutics to the inner ear: The challenge of the blood-labyrinth barrier. *Science Translational Medicine*.

[29] Liu H., Chen L., Giffen K. P. (2018). Cell-Specific Transcriptome Analysis Shows That Adult Pillar and Deiters' Cells Express Genes Encoding Machinery for Specializations of Cochlear Hair Cells. *Frontiers in Molecular Neuroscience*.

[30] Shi X. (2016). Pathophysiology of the cochlear intrastrial fluid-blood barrier (review). *Hearing Research*.

[31] Chen P., Chai Y., Liu H. (2018). Postnatal Development of Microglia-Like Cells in Mouse Cochlea. *Neural Plasticity*.

[32] Zdebik A. A., Wangemann P., Jentsch T. J. (2009). Potassium ion movement in the inner ear: insights from genetic disease and mouse models. *Physiology*.

[33] Jin Z., Uhlen I., Wei-Jia K., Mao-li D. (2009). Cochlear homeostasis and its role in genetic deafness. *Journal of Otology*.

[34] Lang F., Vallon V., Knipper M., Wangemann P. (2007). Functional significance of channels and transporters expressed in the inner ear and kidney. *American Journal of Physiology-Cell Physiology*.

[35] Egilmez O. K., Kalcioglu M. T. (2016). Genetics of Nonsyndromic Congenital Hearing Loss. *Scientifica*.

[36] Marcus D. C., Wu T., Wangemann P., Kofuji P. (2002). KCNJ10 (Kir4.1) potassium channel knockout abolishes endocochlear potential. *American Journal of Physiology-Cell Physiology*.

[37] Liu H., Li Y., Chen L. (2016). Organ of Corti and Stria Vascularis: Is there an Interdependence for Survival?. *PLoS One*.

[38] Goding C. R., Arnheiter H. (2019). MITF-the first 25 years. *Genes & Development*.

[39] Chen T., Zhao B., Liu Y. (2018). MITF-M regulates melanogenesis in mouse melanocytes. *Journal of Dermatological Science*.

[40] Michael H. T., Graff-Cherry C., Chin S. (2018). Partial Rescue of Ocular Pigment Cells and Structure by Inducible Ectopic Expression of Mitf-M in MITF-Deficient Mice. *Investigative Opthalmology & Visual Science*.

[41] Locher H., de Groot J. C. M. J., van Iperen L., Huisman M. A., Frijns J. H. M., Chuva de Sousa Lopes S. M. (2015). Development of the stria vascularis and potassium regulation in the human fetal cochlea: Insights into hereditary sensorineural hearing loss. *Developmental Neurobiology*.

[42] Chen J., Zhao H. B. (2014). The role of an inwardly rectifying K^+^ channel (Kir4.1) in the inner ear and hearing loss. *Neuroscience*.

[43] Yang H., Xiong H., Huang Q. (2013). Compromised potassium recycling in the cochlea contributes to conservation of endocochlear potential in a mouse model of age-related hearing loss. *Neuroscience Letters*.

[44] Wangemann P., Itza E. M., Albrecht B. (2004). Loss of KCNJ10 protein expression abolishes endocochlear potential and causes deafness in Pendred syndrome mouse model. *BMC Medicine*.

[45] Mittal R., Aranke M., Debs L. H. (2017). Indispensable role of ion channels and transporters in the auditory system. *Journal of Cellular Physiology*.

[46] Miller A. J., Du J., Rowan S., Hershey C. L., Widlund H. R., Fisher D. E. (2004). Transcriptional regulation of the melanoma prognostic marker melastatin (TRPM1) by MITF in melanocytes and melanoma. *Cancer Research*.

[47] Inagaki K., Suzuki T., Ito S. (2006). Oculocutaneous albinism type 4: six novel mutations in the membrane-associated transporter protein gene and their phenotypes. *Pigment Cell Research*.

[48] Hoek K. S., Schlegel N. C., Eichhoff O. M. (2008). Novel MITF targets identified using a two-step DNA microarray strategy. *Pigment Cell & Melanoma Research*.

[49] Du J., Fisher D. E. (2001). Identification ofAim-1as theunderwhiteMouse Mutant and Its Transcriptional Regulation by MITF. *Journal of Biological Chemistry*.

[50] Dolga A. M., Culmsee C. (2012). Protective roles for potassium SK/KCa2 channels in microglia and neurons. *Frontiers in Pharmacology*.

[51] Wilms V., Köppl C., Söffgen C., Hartmann A.-M., Nothwang H. G. (2016). Molecular bases of K^+^ secretory cells in the inner ear: shared and distinct features between birds and mammals. *Scientific Reports*.

[52] Strutz-Seebohm N., Seebohm G., Fedorenko O. (2006). Functional Coassembly of KCNQ4 with KCNE-*ß*- Subunits in Xenopus Oocytes. *Cellular Physiology and Biochemistry*.

[53] Bartle E. I., Rao T. C., Urner T. M., Mattheyses A. L. (2017). Bridging the gap: Super-resolution microscopy of epithelial cell junctions. *Tissue Barriers*.

[54] Liu W., Schrott-Fischer A., Glueckert R., Benav H., Rask-Andersen H. (2017). The Human "Cochlear Battery" - Claudin-11 Barrier and Ion Transport Proteins in the Lateral Wall of the Cochlea. *Frontiers in Molecular Neuroscience*.

[55] Kitajiri S., Katsuno T., Sasaki H., Ito J., Furuse M., Tsukita S. (2014). Deafness in occludin-deficient mice with dislocation of tricellulin and progressive apoptosis of the hair cells. *Biology Open*.

[56] Wallez Y., Huber P. (2008). Endothelial adherens and tight junctions in vascular homeostasis, inflammation and angiogenesis. *Biochimica et Biophysica Acta (BBA) - Biomembranes*.

[57] Liu Y., Qi J., Chen X. (2019). Critical role of spectrin in hearing development and deafness. *Science Advances*.

[58] Goncharov N. V., Nadeev A. D., Jenkins R. O., Avdonin P. V. (2017). Markers and biomarkers of endothelium: when something is rotten in the state. *Oxidative Medicine and Cellular Longevity*.

[59] Trune D. R. (2010). Ion homeostasis in the ear: mechanisms, maladies, and management. *Current Opinion in Otolaryngology & Head and Neck Surgery*.

[60] Zhang Y., Guo L., Lu X. (2018). Characterization of Lgr6+ Cells as an Enriched Population of Hair Cell Progenitors Compared to Lgr5+ Cells for Hair Cell Generation in the Neonatal Mouse Cochlea. *Frontiers in Molecular Neuroscience*.

[61] Zhang S., Zhang Y., Yu P. (2017). Characterization of Lgr5+ progenitor cell transcriptomes after neomycin injury in the neonatal mouse cochlea. *Frontiers in Molecular Neuroscience*.

[62] Cheng C., Guo L., Lu L. (2017). Characterization of the transcriptomes of Lgr5+ hair cell progenitors and Lgr5- supporting cells in the mouse cochlea. *Frontiers in Molecular Neuroscience*.

[63] Gupta R., Dewan I., Bharti R., Bhattacharya A. (2012). Differential expression analysis for RNA-Seq data. *ISRN Bioinformatics*.

[64] Huang D. W., Sherman B. T., Lempicki R. A. (2009). Systematic and integrative analysis of large gene lists using DAVID bioinformatics resources. *Nature Protocols*.

[65] Dennis G., Sherman B. T., Hosack D. A. (2003). DAVID: Database for Annotation, Visualization, and Integrated Discovery. *Genome Biology*.

[66] Zhang S., Zhang Y., Dong Y. (2020). Knockdown of Foxg1 in supporting cells increases the trans-differentiation of supporting cells into hair cells in the neonatal mouse cochlea. *Cellular and Molecular Life Sciences*.

[67] Tang M., Li J., He L. (2019). Transcriptomic profiling of neural stem cell differentiation on graphene substrates. *Colloids and Surfaces B: Biointerfaces*.

[68] Cheng C., Wang Y., Guo L. (2019). Age-related transcriptome changes in Sox2+ supporting cells in the mouse cochlea. *Stem Cell Research & Therapy*.

[69] Van Camp G., Smith R. (2015). http://hereditaryhearingloss.

[70] He Z., Guo L., Shu Y. (2017). Autophagy protects auditory hair cells against neomycin-induced damage. *Autophagy*.

